# Comment on Lan et al. Reducing Diabetic Ketoacidosis in Pediatric Type 1 Diabetes: The Impact of Screening Programs and Early Disease-Modifying Treatment. *J. Clin. Med.* 2026, *15*, 5865

**DOI:** 10.3390/jcm15218556

**Published:** 2026-11-03

**Authors:** Ercan Yılmaz, Zeynep Yamancan Yılmaz

**Affiliations:** 1 Department of Pediatric Allergy and Immunology, Faculty of Medicine, İnönü University, Malatya 44280, Türkiye; yilmaz.ercan@inonu.edu.tr; 2 Department of Pediatric Endocrinology, Faculty of Medicine, İnönü University, Malatya 44280, Türkiye

We read with great interest the review by Lan et al. addressing strategies to reduce diabetic ketoacidosis (DKA) at the diagnosis of pediatric type 1 diabetes (T1D), including public awareness, islet autoantibody screening, structured follow-up, and disease-modifying treatment [1]. Presymptomatic detection represents an important advance in pediatric diabetes care. Two points warrant clarification: screening is the entry point to an integrated screening-to-surveillance pathway, and evidence that immunotherapy delays clinical diabetes should be distinguished from evidence of a reduction in DKA incidence.

The review states that none of the participants in The Environmental Determinants of Diabetes in the Young (TEDDY) study developed DKA at diagnosis [1]. This requires a cohort-specific qualification. Steck et al. compared 43 children diagnosed during prospective TEDDY follow-up with 43 age-matched community controls; none of these 43 TEDDY participants presented with DKA, compared with 14% of controls [2]. This observational comparison associated intensive prospective follow-up with a lower frequency of DKA; it did not establish zero DKA incidence across the entire TEDDY cohort.

A larger TEDDY analysis documented DKA in 23 of 379 children (6.1%) diagnosed with T1D: one of 103 (1.0%) with a first-degree relative with T1D and 22 of 276 (8.0%) without one [3]. Children presenting with DKA were more frequently nonadherent to the study protocol and had longer intervals between their last assessment and diagnosis. The cohort also included children who had withdrawn or been lost to follow-up; not all DKA cases had previously confirmed autoantibody positivity [3]. The family-history difference is particularly relevant to general-population screening programs reaching children without an affected first-degree relative, although it does not establish a causal effect of family history. These observations help clarify the pathway through which screening programs may reduce acute metabolic decompensation.

This pathway comprises distinct but connected components: islet autoantibody screening identifies possible autoimmunity; confirmatory testing and metabolic staging establish disease status; longitudinal metabolic monitoring detects evolving dysglycemia; family education supports symptom recognition; and timely referral and clinical intervention enable treatment when needed. Screening programs may explicitly incorporate all these components. A positive screening result alone is insufficient to prevent DKA without effective longitudinal management. Thus, DKA outcomes should be interpreted in relation to the complete pathway, including monitoring adherence and access to care, rather than attributed to the initial test alone.

A related distinction concerns teplizumab. The primary endpoint of TrialNet TN-10 was the time to clinical T1D in participants with Stage 2 disease: median time to diagnosis was 48.4 months with teplizumab versus 24.4 months with the placebo (hazard ratio, 0.41) [4]. Extended follow-up supported sustained effects on progression and β-cell function [5]. In contrast, PROTECT enrolled children and adolescents with newly diagnosed Stage 3 T1D and assessed change from baseline in stimulated C-peptide at Week 78 as its primary endpoint [6]. Neither trial was designed to establish DKA prevention; PROTECT addressed β-cell preservation after diagnosis rather than DKA at initial presentation. These endpoints demonstrate disease modification, while a reduction in DKA incidence is not yet proven as an independent outcome of teplizumab treatment. A recent pediatric commentary similarly emphasizes realistic expectations and variability in treatment response [7].

Three levels of inference should therefore remain explicit: demonstrated delay of Stage 3 T1D; a plausible opportunity for continued monitoring, education, and more controlled diagnosis during that additional time; and an unproven reduction in DKA incidence attributable to treatment. Additional time does not itself guarantee engagement with care. Screening, surveillance, education, and immunotherapy should be integrated across the care pathway, while their contributions to DKA outcomes are evaluated explicitly. The success of screening programs depends on both identifying islet autoimmunity and maintaining effective follow-up.

## Data Availability

No new data were created or analyzed in this study. Data sharing is not applicable to this article.
